# Regional Gastrointestinal Permeability Patterns in Juvenile Idiopathic Arthritis: A Window into Subclinical Inflammation and Microbiota-Driven Disease Mechanisms

**DOI:** 10.3390/children12121663

**Published:** 2025-12-08

**Authors:** Francesco La Torre, Francesca Marasciulo, Giovanni La Grasta, Vanessa Nadia Dargenio, Stefania Paola Castellaneta, Silvia Amati, Violetta Mastrorilli, Antonella Sisto, Fabio Cardinale, Ruggiero Francavilla, Fernanda Cristofori

**Affiliations:** 1 Department of Paediatrics, Pediatric Rheumatology Center, Giovanni XXIII Paediatric Hospital, University of Bari Aldo Moro, 70126 Bari, Italy; francesco.latorre@policlinico.ba.it (F.L.T.); violetta.mastrorilli@policlinico.ba.it (V.M.); a.sisto2@studenti.unipi.it (A.S.); fabio.cardinale@policlinico.ba.it (F.C.); 2 Interdisciplinary Department of Medicine, Paediatric Section, University of Bari Aldo Moro, 70126 Bari, Italy; f.marasciulo5@studenti.uniba.it (F.M.); g.lagrasta2@studenti.uniba.it (G.L.G.); vanessanadia.dargenio@policlinico.ba.it (V.N.D.); stefaniapaola.castellaneta@policlinico.ba.it (S.P.C.); fernanda.cristofori@policlinico.ba.it (F.C.); 3 Paediatric and Neonatology Unit, Department of Developmental Medicine, San Paolo Hospital, Local Health Authority of Bari, 70123 Bari, Italy; silvia.amati@asl.bari.it

**Keywords:** intestinal permeability, juvenile idiopathic arthritis, mucosal barrier

## Abstract

**Highlights:**

**What are the main findings?**

- JIA patients may show altered gastrointestinal segmental sugar absorption despite globally normal intestinal permeability.

**What are the implication of the main findings?**

- Permeability testing of different gastrointestinal tracts may provide a sensitive tool to uncover hidden mucosal abnormalities that are not captured by global indices.
- The exploratory findings of this study may support a role for the gut–joint axis and microbiota-driven mechanisms in JIA pathogenesis, with potential implications for early diagnosis and therapeutic targeting.

**Abstract:**

**Objectives:** To assess gastrointestinal permeability (GP) in children with Juvenile Idiopathic Arthritis (JIA) using a segment-specific sugar probe approach to assess gastric, small intestinal, and colonic permeability, and to determine whether GP alterations are associated with disease activity. **Methods:** This prospective study included 30 children with JIA and 22 healthy controls who underwent a validated multi-sugar absorption test. Urinary excretion of sucrose, lactulose, mannitol, and sucralose was measured to evaluate gastric, small intestinal, and colonic permeability. All JIA patients had discontinued immunosuppressive therapy for at least three months before testing. None had a relapse of the disease. Disease activity was assessed using the Juvenile Arthritis Disease Activity Score (JADAS10). Comparisons were conducted between patients and controls and between remission and active disease groups. **Results:** None of the participants reported gastrointestinal manifestations. The lactulose/mannitol (LA/MA) ratio, a global index of small intestinal permeability, showed no significant difference between JIA patients and controls, suggesting preserved overall barrier function. However, urinary excretion of lactulose, mannitol, and sucralose was significantly higher in JIA patients, while sucrose excretion was significantly lower, indicating segment-specific alterations in small intestinal, colonic, and gastric permeability. These abnormalities were consistently present, even in patients in clinical remission. No statistically significant differences were observed between remission and active disease groups, though a trend toward increased permeability was noted in the latter. **Conclusions:** Children with JIA exhibit segmental GP alterations that persist independently of clinical disease activity. Despite the relatively small population, this exploratory study suggests subclinical mucosal dysfunction and the need for further investigation into how the gut–joint axis may be playing a role in JIA pathogenesis, including via intestinal microbiota.

## 1. Introduction

The gut microbiota plays a crucial role in maintaining immune homeostasis, and its involvement in rheumatic diseases is becoming increasingly evident. Alterations in microbiota composition can lead to changes in gut permeability (GP), contributing to immune dysregulation and systemic inflammation [1,2,3,4]. When the intestinal barrier is compromised, not only microbial antigens but also bacterial metabolites and signaling molecules can cross or modulate epithelial and immune pathways. This can contribute to systemic immune activation and inflammation beyond mere antigen translocation [5]. This mechanism provides a biologically plausible link between intestinal and articular inflammation.

In this context, the gut–joint axis has emerged as a focus of growing interest, especially in Juvenile Idiopathic Arthritis (JIA). Several studies have proposed that gut dysbiosis may serve as a potential pathogenetic factor in JIA, influencing disease onset and progression by modulating mucosal immunity and systemic inflammatory responses [6,7,8,9]. A growing body of evidence suggests a strong link between disruptions in the gut microbiome and the development of JIA [10]. In fact, early-life practices such as minimizing antibiotic treatments, supporting breastfeeding, and vaginal delivery may offer some protection against the onset of JIA [10]. In particular, some studies have specifically investigated the link between early antibiotic exposure and subsequent JIA, consistently reporting a modest yet significant, dose-dependent increase in risk [11,12,13,14]. Antibiotics, especially when given in the first years of life, can induce lasting alterations in the microbiome, and the loss of beneficial commensal species may impair immune tolerance and increase the risk of autoimmunity [15]. Breastfed infants develop a distinct gut microbiome characterized by higher levels of Bifidobacteria and Lactobacillus, which in turn supports healthier immune maturation [16,17]. Breastfeeding may protect against the risk of some autoimmune diseases, including type I diabetes mellitus, inflammatory bowel disease (IBD), and JIA [18,19]. Other factors that may contribute to JIA onset are oral and fecal microbiomes. The frequent coexistence of gingivitis/periodontitis and inflammatory arthritis points toward a common pattern of immune dysregulation affecting both sites. According to the mucosal origin hypothesis, the immune activity occurring at the oral mucosa may directly contribute to the systemic immune response characteristic of inflammatory arthritis [20]. Oral dysbiosis associated with periodontitis or gingivitis has also been linked to reduced gut microbial diversity. Moreover, conditions such as IBD can present with inflammatory arthritis, further supporting a connection between mucosal immune disturbances and systemic autoimmunity. Several investigations have examined the gut microbiome in JIA using fecal samples to amplify distinct regions of the 16S rRNA gene, consistently showing an association between dysbiosis and the disease [10]. Individuals with JIA also exhibit increased intestinal expression of human leukocyte antigen–DR isotype (HLA-DR), reflecting heightened antigen presentation, together with reduced mRNA expression of key anti-inflammatory mediators [8]. An unbalanced microbiota often results in the overgrowth of Gram-negative bacteria, fostering an environment characterized by oxidative stress, low-grade inflammation, and the generation of pro-inflammatory microbial metabolites such as lipopolysaccharides (LPS) [4]. Compared with healthy controls, JIA patients (oligoarticular and polyarticular) show higher levels of Escherichia coli anti-core lipopolysaccharide (LPS) antibodies and LPS-binding protein, correlating with disease activity score [21].

Although disruption of intestinal homeostasis in autoimmune conditions is increasingly recognized, it remains unclear whether gut dysbiosis acts as a trigger or a consequence of systemic inflammation. Emerging evidence suggests a bidirectional relationship: systemic inflammation can alter the gut environment, promoting the overgrowth of pro-inflammatory taxa, while microbial dysbiosis itself may sustain immune activation through the release of metabolites and cytokine-mediated signaling. This reciprocal interaction may contribute to chronic mucosal imbalance and disease persistence [1,2,3,4,5,6].

Despite these associations, only a limited number of studies have explored the relationship between GP and JIA [22,23,24]. Possibly linking the mucosal and systemic immune reactivity, Picco and colleagues have shown increased gut permeability in JIA based on LA/MA testing compared to controls, similar to findings in RA [22].

Understanding whether alterations in GP correlate with joint inflammation could provide clinically relevant insights. If GP changes precede or accompany disease activity, this would support the view that mucosal barrier dysfunction is integral to the pathogenesis of JIA. In addition, if GP alterations persist during remission, they may indicate a long-lasting or intrinsic mucosal imbalance, potentially amenable to therapeutic intervention.

Considering these considerations, the primary aim of our study was to evaluate GP in patients with JIA by assessing not only global intestinal permeability but also, for the first time in the literature, the segmental gastrointestinal permeability. In this study, the term ‘segmental permeability’ refers to the evaluation of distinct regions of the gastrointestinal tract (stomach, small intestine, and colon) using specific sugar probes (sucrose, lactulose, mannitol, and sucralose) that selectively reflect mucosal integrity at each level.

The secondary aim was to investigate the association between GP alterations and disease activity in our JIA cohort, thereby assessing whether barrier dysfunction correlates with clinical inflammation or persists independently of disease status.

## 2. Materials and Methods

This prospective study included consecutive patients with JIA in different phases of disease activity who were routinely evaluated at the Paediatric Rheumatology Department of Giovanni XXIII Hospital of Bari under a tight control follow-up and standard laboratory tests between 2023 and 2024. Patients were eligible for inclusion if they had a diagnosis of oligoarticular or polyarticular JIA, were between 3 and 18 years old, and had discontinued therapy with non-steroidal anti-inflammatory drugs (NSAIDs for at least three consecutive days), oral corticosteroids, methotrexate (MTX), or biologic agents for at least three months before enrollment. Patients with coexisting organic or functional gastrointestinal diseases, diabetes mellitus, food allergies, or liver disease were excluded. Newly diagnosed patients or patients with active disease were enrolled if they had not received oral therapy before the test. Only steroid joint infiltrations were allowed before the test.

For each patient enrolled in the study, written informed consent was obtained from parents or legal guardians. The protocol was approved by the Institutional Ethical Committee (Approval Number: 7612 of 2 February 2023).

GP was assessed using the Gastropack Mass-Q test according to manufacturer instructions (Supplementary Materials) [25]. One week prior to testing, patients were instructed to avoid any use of probiotics. On the day before the test, consumption of sweet foods was not permitted. The evening meal before the test consisted of meat or fish, with no intake of carbohydrates or dairy products. After dinner, patients were allowed to drink only water. On the day of the test, a baseline urine sample was collected. Subsequently, each patient ingested an oral solution composed of water containing four sugar probes: 5 g of lactulose, 1 g of mannitol, 20 g of sucrose, and 1 g of sucralose. Six hours after ingestion of the sugar solution, a second urine sample was collected. During the entire test period, patients remained fasting but were required to drink at least 500 mL of water. The urinary concentrations of the ingested sugars were analyzed. Although reference values have been reported in the literature [26], they are available only for some parameters, such as lactulose and mannitol, and are based on a small number of subjects. Therefore, we enrolled our own control population to provide a more reliable comparison for the study results.

Lactulose absorption reflects small intestinal mucosal integrity, while mannitol absorption indicates villous surface area and trans-epithelial permeability. Both sugars are inert and non-metabolizable and are excreted entirely in the urine within six hours of ingestion, making them ideal probes for evaluating small bowel function [22,23]. Under physiological conditions, lactulose is absorbed at very low levels (<1%); elevated urinary excretion is considered indicative of increased intestinal permeability [22,23]. The LA/MA ratio, a composite index of small intestinal permeability, is the standard reference for assessing global intestinal barrier function [22,23,25]. Each probe targets a different gastrointestinal segment, and absolute recoveries were analyzed when ratio data were unavailable [27,28,29].

The same day, after the collection of the baseline urine sample and ingestion of the oral solution, a rheumatological assessment was performed and disease activity was evaluated using the JADAS10 score. This validated tool categorizes JIA activity into four distinct states (remission, low, moderate, and high disease activity) based on the score obtained during the clinical visit [30]. All patients underwent a detailed clinical assessment, including evaluation for gastrointestinal symptoms such as abdominal pain, diarrhea, bloating, or altered bowel habits. During the clinical evaluation, a detailed medical history was collected, with particular attention to the use of any medications in the previous month.

The control group consisted of healthy children, evaluated for “functional joint pain”, who underwent standard routine laboratory tests, including erythrocyte sedimentation rate and C-reactive protein, all within normal ranges, showing no evidence of inflammation or organic disease.

These individuals underwent the same intestinal permeability test under identical conditions and exhibited no clinical or laboratory evidence of inflammatory disease.

### Statistical Analysis

The level of statistical significance (alpha) was set at 0.05. Continuous variables were expressed as mean ± standard deviation (SD) or interquartile range as appropriate, while categorical variables were reported as frequencies and percentages. Group comparisons for continuous variables were initially performed using parametric analysis of variance (ANOVA). When appropriate, non-parametric tests, including the Mann–Whitney test and the Kruskal–Wallis test, were applied to assess differences between groups. Associations between categorical variables were analyzed using the chi-square test. Correlations between continuous variables were evaluated using Spearman’s rank correlation coefficient (Spearman’s rho, r), to account for potential non-linear relationships.

## 3. Results

A total of 30 patients with JIA were enrolled in the study, comprising 16 females (53%) and 14 males (47%). The control group included 22 healthy subjects (10 males and 12 females). All participants successfully completed the GP test. The age of participants ranged from 4 to 17 years, with a mean age of 11.3 years.

Among the JIA patients, 25 were diagnosed with the oligoarticular subtype and 5 with the polyarticular subtype. At the time of evaluation, 26 patients (87%) were in clinical remission, while 4 patients (13%) had active disease, 2 with moderate and 2 with low disease activity, according to the JADAS10 scoring system. The mean JADAS10 score across the cohort was 0.89, consistent with a remission state. Mean disease duration was 68.3 months (range: 6–168 months).

All patients had previously been treated with NSAIDs during the early phase of disease, either prior to diagnosis or during disease flares. Nine patients had received oral corticosteroids due to a polyarticular course, inadequate response to methotrexate (MTX), or prior to initiating biologic therapy. Eleven patients were treated with MTX, either for a polyarticular course or following insufficient response to intra-articular steroid injections. Two patients required biologic therapy due to lack of response to MTX, one with etanercept and one with adalimumab. All JIA patients were off any immunosuppressive treatment for at least three months before undergoing the GP test. Timing of therapy discontinuation was 34.9 months (range: 4–81 months). All patients did not have any oral therapy in the last 3 months (NSAIDs for at least three consecutive days, oral steroids, MTX, or biologics). None of the participants reported overt gastrointestinal manifestations at the time of the study.

Demographic data for both patients and controls are presented in Table 1.

**Figure 1:**
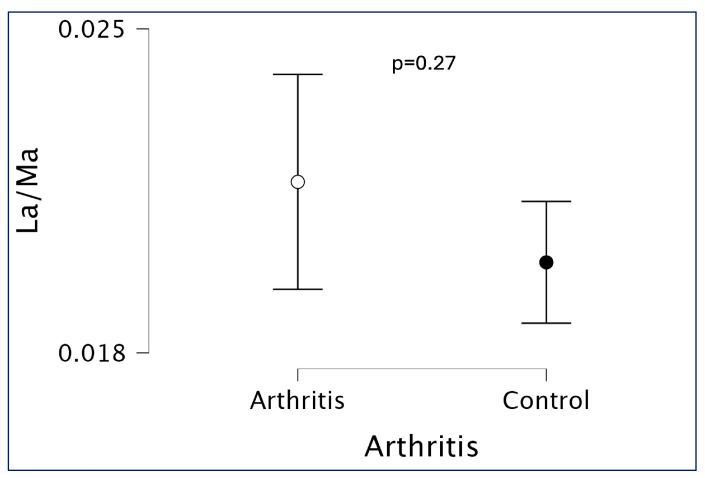
Intestinal permeability in JIA patients assessed by the lactulose/mannitol (LA/MA) ratio, stratified by disease activity. No significant difference was observed between patients in remission and those with active disease. Both groups demonstrated values within the normal range, consistent with preserved gut barrier function. Mean values and interquartile range.

**Figure 2:**
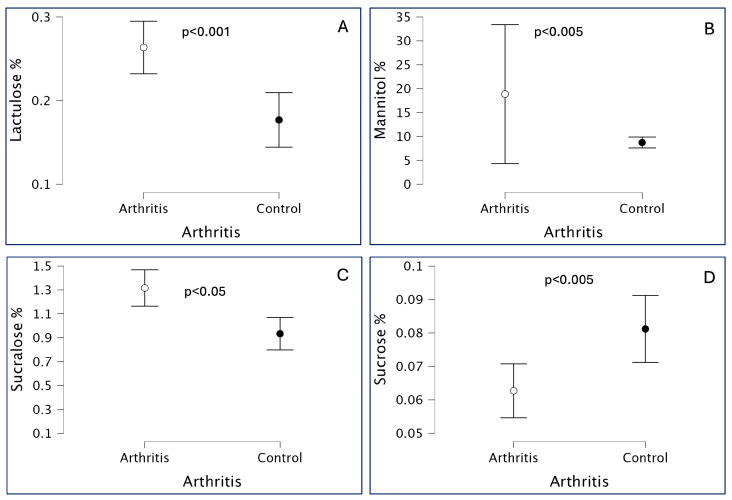
Intestinal absorption profiles of the four sugar probes in patients with Juvenile Idiopathic Arthritis (JIA) compared to healthy controls. Urinary excretion of lactulose (**A**), mannitol (**B**), and sucralose (**C**) was significantly increased in the JIA group, indicating enhanced permeability of the small intestinal mucosa, villous surface, and colonic mucosa, respectively. In contrast, sucrose excretion (**D**), reflecting gastric permeability, was significantly reduced in JIA patients relative to controls. These findings suggest a complex and segment-specific alteration of gastrointestinal barrier function in JIA. Mean values and interquartile range (lactulose and mannitol), mean values and SD (sucralose and sucrose).

When assessing overall small intestinal permeability using the (LA/MA) ratio. No significant differences were found between JIA patients and healthy controls, suggesting normal GP in the JIA group (Table 2, Figure 1). Analysis of individual sugar absorption revealed significant abnormalities in the JIA group compared to controls (Figure 2). Specifically, JIA patients exhibited increased urinary excretion of lactulose, reflecting altered permeability of the small intestinal mucosa (Figure 2A); increased mannitol excretion, indicative of changes in villous absorption (Figure 2B); and elevated sucralose excretion, consistent with increased colonic permeability (Figure 2C). In contrast, sucrose excretion, which reflects gastric permeability, was significantly reduced in JIA patients compared to healthy controls (Figure 2D). Table S1 reports the effect sizes (Cohen’s d) and the corresponding 95% confidence intervals.

For the LA/MA ratio, which did not differ significantly between groups (U = 389.5, *p* = 0.274, rank-biserial correlation rrb* = −0.18, 95% CI −0.46 to 0.14), a post hoc power analysis based on the observed effect size (Cohen’s d = 0.34) and sample sizes (30 patients, 22 controls) indicated an achieved power of only 22% (two-sided α = 0.05), suggesting that this non-significant finding may largely reflect limited statistical power rather than the absence of any between-group difference.

**Figure 3:**
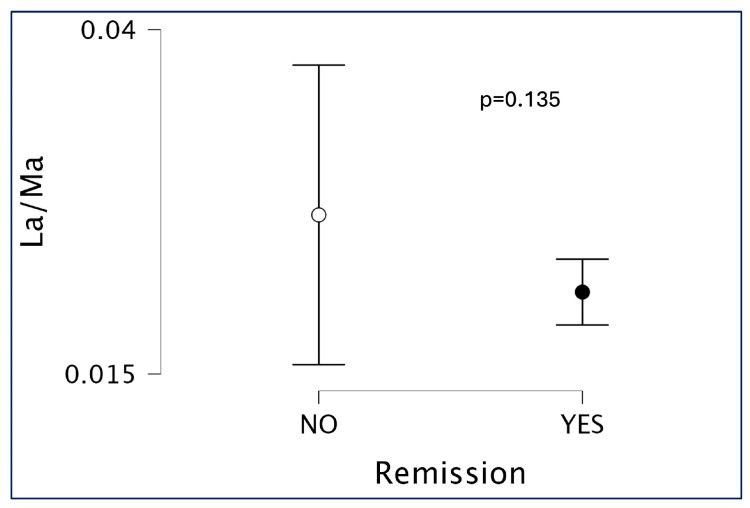
Lactulose/mannitol (LA/MA) ratio in patients with Juvenile Idiopathic Arthritis (JIA), stratified by disease activity status. Remission disease activity is defined as JADAS10 <1.4 for oligoarticular patients or JADAS10 <2.7 in the case of polyarticular patients. Patients in remission exhibited a slightly higher LA/MA ratio compared to those with active disease, although the difference was not statistically significant. These findings suggest that overall intestinal permeability, as measured by the LA/MA ratio, remains relatively stable regardless of disease activity. Mean values and interquartile range.

**Figure 4:**
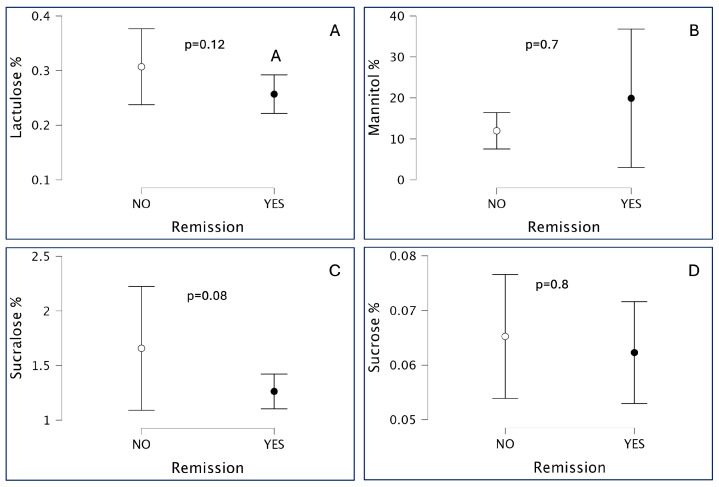
Urinary excretion of the four sugar probes in patients with Juvenile Idiopathic Arthritis (JIA), stratified by disease activity status. Remission disease activity is defined asJADAS10 <1.4 for oligoarticular patients or JADAS10 <2.7 in the case of polyarticular patients. No statistically significant differences were observed between patients in remission and those with active disease for lactulose (**A**), mannitol (**B**), sucralose (**C**), or sucrose (**D**). Mean values and interquartile range (lactulose and mannitol), mean values and SD (sucralose and sucrose).

When stratifying patients by disease activity, a trend toward altered intestinal permeability was observed in patients with active disease compared to those in remission, although this difference did not reach statistical significance (*p* = 0.09; Figure 3). Further analysis of individual sugar absorption by disease activity status did not reveal any statistically significant differences for lactulose, mannitol, sucralose, or sucrose between patients in remission and those with active disease (Figure 4).

## 4. Discussion

The association between gastrointestinal tract inflammation and joint involvement is well established. However, data regarding alterations in the intestinal mucosal barrier and the specific characteristics of intestinal inflammation in JIA remain limited [6,7,8,9].

Mielants et al. first demonstrated the relevance of intestinal inflammation in JIA, reporting histological evidence of colonic and terminal ileum inflammation in 9 out of 12 patients (75%) undergoing colonoscopy for late-onset juvenile chronic arthritis [31]. Kokkonen et al. further highlighted immune activation in the gastrointestinal mucosa of children with JIA or connective tissue diseases, identifying lymphoid nodular hyperplasia and increased CD3+ lymphocyte infiltration as key findings in children presenting with gastrointestinal symptoms [32]. Arvonen et al. observed an elevated number of γδ intraepithelial lymphocytes in JIA patients, suggesting an abnormal cytotoxic response within the intestinal mucosa [33]. In another histopathological study, Pichler et al. documented mucosal inflammation in 85% of JIA patients undergoing biopsy for gastrointestinal symptoms, with eosinophilia present in one-third of cases, suggesting a complex interplay between immune dysregulation and intestinal epithelial integrity [34].

The integration of these findings with our results points to a model in whichsubclinical mucosal immune dysregulation and microbiota-driven barrier dysfunctionpersist in JIA, contributing to disease pathogenesis. We aimed to further characterize mucosal alterations in JIA by assessing segment-specific GP. To our knowledge, this is the first study evaluating not only small intestinal permeability via the LA/MA ratio but also region-specific permeability in the stomach, small intestine, and colon using individual sugar probes.

The intestinal permeability test presents some limitations. It is time-consuming, and several confounding factors, such as differences in intestinal motility, mucosal surface area, epithelial integrity, renal function, bacterial degradation, gastric dilution, and diet, may influence the interpretation of results. Moreover, the absence of standardized protocols contributes to variability across studies. Hence, the results should be viewed with caution in light of these methodological limitations and potential confounding factors. As suggested by recent reports, greater uniformity in probe selection, test duration, and fasting conditions may help reduce such inconsistencies [35].

In our cohort, although the LA/MA ratio did not differ significantly between JIA patients and healthy controls (Figure 1), suggesting preserved overall small intestinal permeability, a post hoc power analysis showed an achieved power of only 22%. Therefore, this non-significant finding should be interpreted with caution, as it may largely reflect limited statistical power rather than the absence of a true between-group difference. We observed significantly elevated urinary recovery of both lactulose (Figure 2A) and mannitol (Figure 2B) in JIA patients, even in clinical remission, indicating subclinical alterations in small intestinal permeability. The increased mannitol absorption likely compensates for the elevated lactulose absorption, thus maintaining a normal LA/MA ratio. These findings imply that despite remission, mucosal barrier abnormalities persist in JIA.

Our results are in partial agreement with Picco et al., who reported increased intestinal permeability in a cohort of JIA patients using the LA/MA ratio measured by gas chromatography. However, most of their cohort had active disease and ongoing NSAID treatment, potentially contributing to the observed changes [22]. In contrast, our study included only patients who had been off therapy for at least three months, thereby minimizing pharmacological confounding. Nevertheless, previous exposure to anti-inflammatory or immunomodulatory agents may have had residual effects on mucosal permeability that cannot be entirely excluded.”

Although we observed a trend toward increased intestinal permeability in patients with active disease (Figure 3), this association did not reach statistical significance (*p* = 0.09), likely due to the limited number of patients in the active disease group (n = 4). Still, our findings support the hypothesis that mucosal barrier disruption may be more pronounced during disease flares.

The influence of NSAIDs on gastrointestinal permeability is well-documented. Nevertheless, Weber et al. found no significant difference in LA/MA ratios between NSAID-treated JIA patients and healthy controls, suggesting that not all NSAID regimens result in measurable permeability alterations [23]. Our study controlled for this variable by excluding patients with recent NSAID exposure, further strengthening the reliability of our findings.

Interestingly, our JIA cohort also demonstrated significantly increased sucralose absorption (Figure 2C), suggesting altered colonic permeability even in remission. This novel finding, not previously described in JIA literature, indicates persistent colonic mucosal abnormalities, further supporting the hypothesis of subclinical gastrointestinal involvement.

Conversely, we observed significantly reduced sucrose absorption in JIA patients compared to controls (Figure 2D), indicating impaired gastric permeability. This could reflect residual functional damage to the gastric mucosa from previous use of NSAIDs, corticosteroids, MTX, or biologic therapies—even though all patients had been off treatment for at least three months. This contrasts with Weber et al., who reported increased sucrose absorption in NSAID-treated JIA patients, likely due to drug-induced gastric mucosal injury and enhanced translocation [23].

Stratification by disease activity revealed no statistically significant differences in absorption of any of the four sugars (Figure 4). However, when considered alongside the elevated absorption of lactulose, mannitol, and sucralose in JIA patients, even in remission, our findings suggest persistent segmental permeability abnormalities despite normalized global gut function.

These results are consistent with emerging literature demonstrating that the gut microbiota can modulate both local and systemic immune responses [36,37]. Notably, studies have shown that commensal bacteria, not only pathogens, may be sufficient to initiate joint inflammation in susceptible hosts [38,39]. In autoimmune conditions, dysbiosis can compromise mucosal integrity, leading to increased permeability and reduced immune tolerance toward commensals [40,41,42,43,44,45,46,47]. Previous studies have indeed identified elevated intestinal permeability in JIA patients [22,48].

The growing body of evidence linking gut microbiota to rheumatic diseases underscores the potential role of lifestyle and dietary habits in shaping microbiota composition and triggering dysbiosis. This, in turn, may promote abnormal sugar absorption, disrupt intestinal permeability, and contribute to systemic immune dysregulation, including synovial inflammation [49,50,51,52].

Crucially, our findings demonstrating altered segmental permeability in JIA (increased lactulose, mannitol, and sucralose absorption) provide a vital physiological foundation for the systemic mechanisms underlying the gut–joint axis. These results align with and are complemented by a recent systematic review and meta-analysis exploring the role of tryptophan-derived microbial metabolites in modulating systemic inflammation and nociception in arthropathies, such as osteoarthritis [53]. The compromised segmental intestinal barrier we observed could therefore represent the pathway through which these altered metabolites or microbial products enter the systemic circulation, potentially contributing to the persistence of inflammation and associated pain in JIA.

Furthermore, the translational potential of these findings is strongly reinforced by literature showing that lifestyle-based interventions (e.g., exercise, probiotics) can effectively modulate the gut microbiome and improve outcomes, including pain levels, in patients with chronic widespread pain [54]. Identifying persistent mucosal barrier abnormalities in JIA, even during remission, suggests that these patients may particularly benefit from targeted therapeutic strategies aimed at restoring intestinal barrier integrity and re-establishing eubiosis.

Our findings suggest that in JIA patients in remission, increased absorption of lactulose, mannitol, and sucralose reflects persistent permeability alterations in the small intestine and colon. However, mannitol hyperabsorption may act as a physiological buffer, maintaining a normal LA/MA ratio despite these abnormalities. We hypothesize that in active disease, mannitol absorption decreases, disrupting this balance and resulting in elevated global GP and systemic immune activation.

This study also has some limitations that should be acknowledged. The sample size was relatively small, particularly in the subgroup of patients with active disease, which limits the strength of statistical comparisons and the generalizability of the findings. For this reason, a direct comparison between patients with active disease and healthy controls could not be performed. The cross-sectional and exploratory design does not allow the establishment of causal relationships between intestinal permeability alterations and disease activity. In addition, potential confounding factors inherent to the intestinal permeability test itself, as previously discussed, cannot be completely ruled out. Therefore, the findings should be considered preliminary and interpreted with due caution.

To validate our hypothesis, future studies with larger cohorts of JIA patients in active disease are warranted.

## 5. Conclusions

In conclusion, this study provides novel insight into the segment-specific alterations of intestinal permeability in patients with JIA, evaluating for the first time the absorption patterns across all major compartments of the gastrointestinal tract. Despite a globally preserved GP profile, JIA patients in clinical remission demonstrated abnormal absorption of lactulose, mannitol, sucralose, and sucrose compared to healthy controls. These findings suggest persistent, subclinical mucosal dysfunction and support the hypothesis that the gut microbiota may act as a key immunological regulator in the pathogenesis and maintenance of JIA, even in the absence of overt inflammation.

The small number of patients with active disease represents a limitation of this study; however, its prospective design and the inclusion of a well-characterized, treatment-free cohort strengthen the validity of the observations. These results underscore the importance of evaluating intestinal function beyond conventional inflammation markers and point to GP testing as a promising tool in understanding immune dysregulation in JIA.

Further studies in larger cohorts, particularly focusing on patients during active disease flares, are essential to validate these findings and to explore the therapeutic implications of targeting gut barrier function and the intestinal microbiota in pediatric rheumatology.

## Figures and Tables

**Table 1:** Baseline Demographic and Clinical Features of Participants.

Characteristic	JIA Patients (n = 30)	Healthy Controls (n = 22)
Age (years)	4.7–17.1 (mean: 11.1)	5.1–17.3 (mean: 11.4)
Female sex	16 (53%)	12 (55%)
Male sex	14 (47%)	10 (45%)
JIA subtype	25 oligoarticular (83%) 5 polyarticular (17%)	—
Disease status	26 in remission (87%) 4 active disease (13%)	—
Mean JADAS10 score	0.89 (indicative of remission)	—
Antibiotic use (30 days before the study)	—	—
Probiotic use (30 days before the study)	—	—
Laxative use (30 days before the study)	—	—
NSAID exposure (prior to study)	All patients	—
Oral corticosteroids (prior use)	9 patients	—
Methotrexate (prior use)	11 patients	—
Biologic therapy (prior use)	2 patients (1 etanercept, 1 adalimumab)	—
Off treatment for ≥3 months	All patients	—

**Table 2:** Segmental Gut Permeability in JIA Patients and Healthy Controls.

Parameter	JIA Patients	Healthy Controls	*p*-Value	Reference Values [26]
Lactulose/Mannitol ratio (LA/MA) Mean (IQR)	0.022 (0.019–0.024)	0.02 (0.018–0.022)	NS	0.024 ± 0.006
Lactulose (%) Mean (IQR)	0.242 (0.196–0.299)	0.165 (0.143–0.198)	<0.001	0.33 ± 0.13
Mannitol (%) Mean (IQR)	18.22 (8.28–31.63)	8.27 (7.44–9.31)	<0.005	14.12 ± 6.63
Sucralose (%) (Mean ± SD)	1.32 ± 0.41	0.93 ± 0.28	<0.005	Not available
Sucrose (%) (Mean ± SD)	0.06 ± 0.022	0.08 ± 0.02	<0.005	Not available
LA/MA—Remission vs. Active	Remission: ~0.030 Active: ~0.023	—	0.09	

## Data Availability

The data that support the findings of this study are not publicly available due to ethical and privacy restrictions involving clinical data. However, anonymized data may be made available from the corresponding author upon reasonable request and with appropriate ethical approval.

## Supplementary Materials

The following supporting information can be downloaded at: https://www.mdpi.com/article/10.3390/children12121663/s1: Characteristics of the permeability test; Supplementary Table S1. Effect sizes (Cohen’s d) and 95% confidence intervals for differences in gastrointestinal permeability parameters between JIA patients and healthy controls.

## Abbreviations

The following abbreviations are used in this manuscript:

GP	Gut permeability
JIA	Juvenile Idiopathic Arthritis
JADAS10	Juvenile Arthritis Disease Activity Score (10-joint count version)
NSAIDs	Non-Steroidal Anti-Inflammatory Drugs
MTX	Methotrexate
LA/MA	Lactulose/Mannitol ratio
SD	Standard Deviation
ANOVA	Analysis of Variance
LPS	Lipopolysaccharides

## References

[1] Juárez-Chairez M.F., Cid-Gallegos M.S., Jiménez-Martínez C., Prieto-Contreras L.F., Bollain-Y-Goytia de-la-Rosa J.J. (2024). The role of microbiota on rheumatoid arthritis onset. Int. J. Rheum. Dis..

[2] Gilbert B.T.P., Tadeo R.Y.T., Lamacchia C., Studer O., Courvoisier D., Raes J., Finckh A. (2024). Gut microbiome and intestinal inflammation in preclinical stages of rheumatoid arthritis. RMD Open.

[3] Yang Y., Hong Q., Zhang X., Liu Z. (2024). Rheumatoid arthritis and the intestinal microbiome: Probiotics as a potential therapy. Front. Immunol..

[4] Wu R., Wang D., Cheng L., Su R., Li B., Fan C., Gao C., Wang C. (2024). Impaired immune tolerance mediated by reduced Tfr cells in rheumatoid arthritis linked to gut microbiota dysbiosis and altered metabolites. Arthritis Res. Ther..

[5] Kuhn K.A., Yomogida K., Knoop K., Wu H.-J.J., Zaiss M.M. (2025). More than a leaky gut: How gut priming shapes arthritis. Nat. Rev. Rheumatol..

[6] Stoll M.L., Cron R.Q. (2016). The microbiota in pediatric rheumatic disease: Epiphenomenon or therapeutic target?. Curr. Opin. Rheumatol..

[7] Xin L., He F., Li S., Zhou Z.-X., Ma X.-L. (2020). Intestinal microbiota and juvenile idiopathic arthritis: Current understanding and future prospective. World J. Pediatr..

[8] Fotis L., Shaikh N., Baszis K.W., Samson C.M., Lev-Tzion R., French A.R., Tarr P.I. (2017). Serologic Evidence of Gut-driven Systemic Inflammation in Juvenile Idiopathic Arthritis. J. Rheumatol..

[9] De Filippo C., Di Paola M., Giani T., Tirelli F., Cimaz R. (2019). Gut microbiota in children and altered profiles in juvenile idiopathic arthritis. J. Autoimmun..

[10] Cole L.D., Kuhn K.A. (2025). It Takes a Village: Juvenile Idiopathic Arthritis and the Microbiome. Rheum. Dis. Clin. N. Am..

[11] Horton D.B., Scott F.I., Haynes K., Putt M.E., Rose C.D., Lewis J.D., Strom B.L. (2015). Antibiotic Exposure and Juvenile Idiopathic Arthritis: A Case–Control Study. Pediatrics.

[12] Arvonen M., Virta L.J., Pokka T., Kröger L., Vähäsalo P. (2015). Repeated exposure to antibiotics in infancy: A pre-disposing factor for juvenile idiopathic arthritis or a sign of this group’s greater susceptibility to infections?. J. Rheumatol..

[13] Hestetun S., Andersen S., Sanner H., Størdal K. (2023). Antibiotic exposure in prenatal and early life and risk of juvenile idiopathic arthritis: A nationwide register-based cohort study. RMD Open.

[14] Kindgren E., Ahrens A.P., Triplett E.W., Ludvigsson J. (2023). Infant gut microbiota and environment associate with juvenile idiopathic arthritis many years prior to disease onset, especially in genetically vulnerable children. EBioMedicine.

[15] Korpela K., Salonen A., Virta L.J., Kekkonen R.A., de Vos W.M. (2016). Association of early-life antibiotic use and protective effects of breastfeeding: Role of the intestinal microbiota. JAMA Pediatr..

[16] Harmsen H.J., Wildeboer–Veloo A.C., Raangs G.C., Wagendorp A.A., Klijn N., Bindels J.G., Welling G.W. (2000). Analysis of intestinal flora development in breast-fed and formula-fed infants by using molecular identifi cation and detection methods. J. Pediatr. Gastroenterol. Nutr..

[17] Rautava S., Luoto R., Salminen S., Isolauri E. (2012). Microbial contact during pregnancy, intestinal colonization and human disease. Nat. Rev. Gastroenterol. Hepatol..

[18] Hummel S., Weiß A., Bonifacio E., Agardh D., Akolkar B., Aronsson C.A., Hagopian W.A., Koletzko S., Krischer J.P., Lernmark Å. (2021). Associations of breastfeeding with childhood autoimmunity, allergies, and overweight: The Environmental Determinants of Diabetes in the Young (TEDDY) study. Am. J. Clin. Nutr..

[19] Hashash J.G., Elkins J., Lewis J.D., Binion D.G. (2024). AGA Clinical Practice Update on Diet and Nutritional Therapies in Patients with Inflammatory Bowel Disease: Expert Review. Gastroenterology.

[20] Holers V.M., Demoruelle M.K., Kuhn K.A., Buckner J.H., Robinson W.H., Okamoto Y., Deane K. (2018). D Rheumatoid arthritis and the mucosal origins hypothesis: Protection turns to destruction. Nat. Rev. Rheumatol..

[21] Arvonen M., Vähäsalo P., Turunen S., Salo H.M., Mäki M., Laurila K., Karttunen T.J. (2012). Altered expression of intestinal human leu-cocyte antigen D-related and immune signalling molecules in juvenile idio pathic arthritis. Clin. Exp. Immunol..

[22] Picco P., Gattorno M., Marchese N., Vignola S., Sormani M.P., Barabino A., Buoncompagni A. (2001). Increased gut permeability in juvenile chronic arthritides. A multivariate analysis of the diagnostic parameters. Clin. Exp. Rheumatol..

[23] Weber P., Brune T., Ganser G., Zimmer K.P. (2003). Gastrointestinal symptoms and permeability in patients with juvenile idiopathic 364 arthritis. Clin. Exp. Rheumatol..

[24] Audo R., Sanchez P., Rivière B., Mielle J., Tan J., Lukas C., Macia L., Morel J., Daien C.I. (2022). Rheumatoid arthritis is associated with increased gut permeability and bacterial translocation that are reversed by inflammation control. Rheumatology.

[25] Sarotra P., Dutta U., Gupta H., Kartha K.P.R., Kochhar R., Prakash A., Sarma P., Shah J., Medhi B. (2022). Simultaneous 366 determination of lactulose, sucrose, sucralose, and mannitol using high-performance liquid chromatography-refractive index 367 to estimate intestinal permeability in patients with active ulcerative colitis. Indian. J. Pharmacol..

[26] Marsilio R., D’aNtiga L., Zancan L., Dussini N., Zacchello F. (1998). Simultaneous HPLC determination with light-scattering detection of lactulose and mannitol in studies of intestinal permeability in pediatrics. Clin. Chem..

[27] Bjarnason I. (1994). Intestinal permeability. Gut.

[28] Lostia A.M., Lionetto L., Principessa L., Evangelisti M., Gamba A., Villa M.P., Simmaco M. (2008). A liquid chromatography/mass spectrometry method for the evaluation of intestinal permeability. Clin. Biochem..

[29] Bischoff S.C., Barbara G., Buurman W., Ockhuizen T., Schulzke J.-D., Serino M., Tilg H., Watson A., Wells J.M., Pihlsgård M. (2014). Intestinal permeability—A new target for disease prevention and therapy. BMC Gastroenterol..

[30] Trincianti C., Van Dijkhuizen E.H.P., Alongi A., Mazzoni M., Swart J.F., Nikishina I., Lahdenne P., Rutkowska-Sak L., Avcin T., Quartier P. (2021). Definition and Validation of the American College of Rheumatology 2021 Juvenile Arthritis Disease Activity Score Cutoffs for Disease Activity States in Juvenile Idiopathic Arthritis. Arthritis Rheumatol..

[31] Mielants H., Veys E.M., Cuvelier C., De Vos M., Goemaere S., Maertens M., Joos R. (1993). Gut inflammation in children with late onset pauciarticular juvenile chronic arthritis and evolution to adult spondyloarthropathy—A prospective study. J. Rheumatol..

[32] Kokkonen J., Arvonen M., Vähäsalo P., Karttunen T.J. (2007). Intestinal immune activation in juvenile idiopathic arthritis and connective tissue disease. Scand. J. Rheumatol..

[33] Arvonen M., Berntson L., Pokka T., Karttunen T.J., Vähäsalo P., Stoll M.L. (2016). Gut microbio-ta-host interactions and juvenile idiopathic arthritis. Pediatr. Rheumatol. Online J..

[34] Pichler J., Ong C., Shah N., Sebire N., Kiparrissi F., Borrelli O., Pilkington C., Elawad M. (2016). Histopathological features of gastrointestinal mucosal biopsies in children with juvenile idiopathic arthritis. Pediatr. Res..

[35] Galipeau H.J., Verdu E.F. (2016). The complex task of measuring intestinal permeability in basic and clinical science. Neurogastroenterol. Motil..

[36] Chung H., Kasper D.L. (2010). Microbiota-stimulated immune mechanisms to maintain gut homeostasis. Curr. Opin Immunol..

[37] Stoll M.L., Kumar R., Morrow C.D., Lefkowitz E.J., Cui X., Genin A., Cron R.Q., Elson C.O. (2014). Altered microbiota associated with abnormal humoral immune responses to commensal organisms in enthesitis-related arthritis. Arthritis Res. Ther..

[38] Rath H.C., Herfarth H.H., Ikeda J.S., Grenther W.B., Hamm T.E., Balish E., Taurog J.D., Hammer R.E., Wilson K.H., Sartor R.B. (1996). Normal luminal bacteria, especially Bacteroides species, mediate chronic colitis, gastritis, and arthritis in HLA-B27/human beta2 microglobulin transgenic rats. J. Clin. Investig..

[39] Abdollahi-Roodsaz S., Joosten L.A., Helsen M.M., Walgreen B., van Lent P.L., van den Bersselaar L.A., Koenders M.I., van den Berg W.B. (2008). Shift from toll-like receptor 2 (TLR-2) to-ward TLR-4 dependency in the erosive stage of chronic streptococcal cell wall arthritis coincident with TLR-4-mediated interleukin-17 production. Arthritis Rheum..

[40] Longman R.S., Littman D.R. (2015). The functional impact of the intestinal microbiome on muco-sal immunity and systemic autoimmunity. Curr. Opin. Rheumatol..

[41] Brusca S.B., Abramson S.B., Scher J.U. (2014). Microbiome and mucosal inflammation as ex-tra-articular triggers for rheumatoid arthritis and autoimmunity. Curr. Opin. Rheumatol..

[42] Costello M.E., Ciccia F., Willner D., Warrington N., Robinson P.C., Gardiner B., Marshall M., Kenna T.J., Triolo G., Brown M.A. (2015). Brief Report: Intestinal Dysbiosis in Ankylosing Spondylitis. Arthritis Rheumatol..

[43] Ubeda C., Bucci V., Caballero S., Djukovic A., Toussaint N.C., Equinda M., Lipuma L., Ling L., Gobourne A., No D. (2013). Intestinal Microbiota Containing Barnesiella Species Cures Vancomycin-Resistant Enterococcus faecium Colonization. Infect. Immun..

[44] Wells C.L., van de Westerlo E.M., Jechorek R.P., Feltis B.A., Wilkins T.D., Erlandsen S.L. (1996). Bacteroides fragilis enterotoxin modulates epithelial permeability and bacterial internalization by HT-29 enterocytes. Gastroenterology.

[45] Derrien M., Vaughan E.E., Plugge C.M., de Vos W.M. (2004). Akkermansia muciniphila gen. nov., sp. nov., a human intestinal mucin-degrading bacterium. Int. J. Syst. Evol. Microbiol..

[46] Martínez-González O., Cantero-Hinojosa J., Paule-Sastre P., Gómez-Magán J.C., Salvatierra-Ríos D. (1994). Intestinal permeability in patients with ankylosing spondylitis and their healthy relatives. Rheumatology.

[47] Asquith M.J., Stauffer P., Davin S., Mitchell C., Lin P., Rosenbaum J.T. (2016). Perturbed Mucosal Immunity and Dysbiosis Accompany Clinical Disease in a Rat Model of Spondyloarthritis. Arthritis Rheumatol..

[48] Round J.L., Mazmanian S.K. (2009). The gut microbiota shapes intestinal immune responses during health and disease. Nat. Rev. Immunol..

[49] Stamp L.K., James M.J., Cleland L.G. (2005). Diet and rheumatoid arthritis: A review of the literature. Semin. Arthritis Rheum..

[50] Badsha H. (2018). Role of Diet in Influencing Rheumatoid Arthritis Disease Activity. Open Rheumatol. J..

[51] van der Meulen T.A., Harmsen H., Bootsma H., Spijkervet F., Kroese F., Vissink A. (2016). The microbiome-systemic diseases connection. Oral Dis..

[52] Li S., Micheletti R. (2011). Role of Diet in Rheumatic Disease. Rheum. Dis. Clin. N. Am..

[53] Meléndez-Oliva E., Martínez-Pozas O., Sinatti P., Martín Carreras-Presas C., Cuenca-Zaldívar J.N., Turroni S., Sánchez Romero E.A. (2025). Relationship Between the Gut Microbiome, Tryptophan-Derived Metabolites, and Osteoarthritis-Related Pain: A Systematic Review with Meta-Analysis. Nutrients.

[54] Gonzalez-Alvarez M.E., Sanchez-Romero E.A., Turroni S., Fernandez-Carnero J., Villafañe J.H. (2023). Correlation between the Altered Gut Microbiome and Lifestyle Interventions in Chronic Widespread Pain Patients: A Systematic Review. Medicina.

