# Supplementary material for: Regional Gastrointestinal Permeability Patterns in Juvenile Idiopathic Arthritis: A Window into Subclinical Inflammation and Microbiota-Driven Disease Mechanisms
Source: Children (Basel). 2025 Dec 8;12(12):1663. doi: 10.3390/children12121663 (PMC12731787; doi:10.3390/children12121663)
Supplement: Supplementary file 1 [file children-12-01663-s001.zip › children-3899041-supplementary.pdf]

Supplementary material

1. Characteristics of the permeability test

1.1 Sample preparation

Stock solutions are prepared at concentrations of 1 mg/mL in water. Working solutions are prepared through serial dilutions in water for all 4 substrates. For each sugar, 7-point calibration curves are prepared with the following concentration ranges, expressed in µg/mL: Sucrose (2.5-100); Lactulose (2.5-200); Mannitol (10-1000); Sucralose (1-100); each analytical session includes quality controls at the beginning and end of the sequence of the calibration curve for all sugars. The urine samples collected during the 6 hours are stored at room temperature. The simple was first centrifuged at 5000 rpm for 5 minutes and then 50 µL of each sample with its calibrator were transferred into tubes with 200 µL of standard solution and vortexed. The next step involved a 1:5 dilution in Acetonitrile. Once the samples were vortexed, 200 µL of supernatant was transferred into vials for analysis.

1.2 Sample analysis

The UPLC-MS/MS system consists of a Waters ACQUITY UPLC high-performance liquid chromatography interfaced with a Waters TQD (triple quadrupole) mass spectrometer. The chromatographic separation is performed using an ACQUITY UPLC BEH HILIC Column, 130Å, 1.7 µm, 2.1 mm with a single ride lasting 5 minutes. The mobile phases used are Water and Acetonitrile. The oven column temperature is set at 50°C. The sample injection volume is 15 µL. The injection syringe of the autosampler is washed after each injection with a 60% solution of acetonitrile in water. Both quadrupoles are set to work in unit mass resolution. The polarity of the instrument used during the analyzes is electro spray ionization (ESI). The gas used for the collision is Argon. Results are processed through TargetLynx™ software (v 4.1, Waters Corporation). Molecular tuning for each molecule is performed by infusing standard solutions in water at a concentration of 10 µg/mL. In this step, the source parameters were optimized using the Intellistart™ software (Waters Corporation) and the Multiple Reaction Monitoring (MRM) method was created. The analysis method was validated following the EMA guidelines for the validation of a bioanalytical method (EMEA/CHMP/EWP/192217/2009 Rev. 1 Corr. 2** Committee for Medicinal Products for Human Use (CHMP)).

1.3 Analysis reports

The analysis report contains: 1) person information of the patient (Name, Surname, Date of birth, Weight, Height); 2) data relating to the test (Date performed, Substrates administered, Time and Volume of BASE and POST sampling); 3) data analysis (Date analysis, Equation of the calibration lines, Range of the method and Linearity, masses of the 4 analysts present in the BASE sample and in the POST sample); 4)Percentage recoveries of the individual sugars and Reference range. The data relating to the BASE sample are provided as qualitative data to evaluate the situation before the administration of sugars and possibly whether the patient was fasting and followed the test preparation protocol. Recoveries are calculated on the POST sample using the following formula: R% = [Sugar concentration (mg/L) × Urine volume (L) / administered dose (mg)] x 100.

Parameter	Cohen's d	95% CI
**Lactulose** (%)	1.80	[1.15, 2.45]
**Mannitol** (%)	1.28	[0.68, 1.88]
**Sucralose** (%)	1.08	[0.49, 1.67]
**Sucrose** (%)	−0.94	[−1.52, −0.36]

Supplementary Table S1. Effect sizes (Cohen's d) and 95% confidence intervals for differences in gastrointestinal permeability parameters between JIA patients and healthy controls.
